# An EEG-Based Transfer Learning Method for Cross-Subject Fatigue Mental State Prediction

**DOI:** 10.3390/s21072369

**Published:** 2021-03-29

**Authors:** Hong Zeng, Xiufeng Li, Gianluca Borghini, Yue Zhao, Pietro Aricò, Gianluca Di Flumeri, Nicolina Sciaraffa, Wael Zakaria, Wanzeng Kong, Fabio Babiloni

**Affiliations:** 1 School of Computer Science and Technology, Hangzhou Dianzi University, Hangzhou 310018, China; jivon@hdu.edu.cn (H.Z.); lixiufengcn@hdu.edu.cn (X.L.); zhaoyue@hdu.edu.cn (Y.Z.); kongwanzeng@hdu.edu.cn (W.K.); 2 Key Laboratory of Brain Machine Collaborative Intelligence of Zhejiang Province, Hangzhou 310018, China; 3 Industrial NeuroScience Lab, University of Rome “La Sapienza”, 00161 Rome, Italy; gianluca.borghini@uniroma1.it (G.B.); pietro.arico@uniroma1.it (P.A.); gianluca.diflumeri@uniroma1.it (G.D.F.); nicolina.sciaraffa@uniroma1.it (N.S.); 4 Department of Mathematics-Computer Science, Faculty of Science, Ain Shams University, Abbassia, Cairo 11435, Egypt; wael.zakaria@sci.asu.edu.eg

**Keywords:** cross-subject prediction, Domain-Adversarial Neural Network (DANN), electroencephalogram (EEG), Generative Adversarial Networks (GAN)

## Abstract

Fatigued driving is one of the main causes of traffic accidents. The electroencephalogram (EEG)-based mental state analysis method is an effective and objective way of detecting fatigue. However, as EEG shows significant differences across subjects, effectively “transfering” the EEG analysis model of the existing subjects to the EEG signals of other subjects is still a challenge. Domain-Adversarial Neural Network (DANN) has excellent performance in transfer learning, especially in the fields of document analysis and image recognition, but has not been applied directly in EEG-based cross-subject fatigue detection. In this paper, we present a DANN-based model, Generative-DANN (GDANN), which combines Generative Adversarial Networks (GAN) to enhance the ability by addressing the issue of different distribution of EEG across subjects. The comparative results show that in the analysis of cross-subject tasks, GDANN has a higher average accuracy of 91.63% in fatigue detection across subjects than those of traditional classification models, which is expected to have much broader application prospects in practical brain–computer interaction (BCI).

## 1. Introduction

Mental fatigue is a complex physiological and psychological state, which can lead to a decline in alertness, concentration and cognitive performance [1]. About 1.3 million people lose their lives in traffic accidents every year in the world [2], and fatigued driving is a leading factor in it [3]. Thus, performing mental state detection and prediction while driving is extremely important to reduce losses of lives and property caused by fatigued driving [4,5].

Numerous fatigued driving detection methods have been proposed. They can be divided into three categories: psychometric questionnaires (e.g., Karolinska Sleepiness Scale (KSS) [6] and Checklist Individual Strength questionnaire (CIS) [7]), behavioral methods (e.g., facial expressions [8], head movement [9], blink rate [10] and eye state [5]) and physiological measurements (e.g., EEG, electrocardiogram (ECG), electrooculogram (EOG) and surface electromyography [8,11,12]). Regarding these questionnaires, they are not only strongly subjective, but they are also unable to monitor the fatigue state in real time, while behavioral methods are extremely susceptible to interference from the road environment, resulting in a certain error of judgement. Among physiological measurements, EEG is regarded as the most effective method for mental state detection, since EEG records electric activities of neural cells from the human cerebral cortex, which can directly reflect instant states of the brain and avoid the effects of human subjectivity [10,13].

Most existing EEG-based methods for fatigued driving detection focus on extracting suitable features and designing classifiers. Chai et al. used power spectral density (PSD) as the feature extraction method and Bayesian neural networks (BNN) as the classifier for classifying alert and fatigue [14]. Rahma et al. extracted the Common Spatial Pattern (CSP) feature to train extreme learning machine (ELM) for fatigue detection [15]. Huo et al. [16] combined EEG and forehead EOG to detect fatigue level of drivers by using a discriminative graph regularized extreme learning machine (GELM). San et al. [17] proposed a hybrid deep generic model (DGM)-based support vector machine for driver fatigue detection. Although effective, such methods mainly focus on EEG analysis under the situation of the same subject, which means the abilities for cross-subject detection are still insufficient.

Nowadays, the domain adaptive-method-based transfer learning models are extensively applied to many fields such as natural language processing and image classification and achieve very good performance. Domain-Adversarial Neural Network (DANN) [18] is a typical domain adaptive method, and the most important feature of DANN is to align the source domain and target domain without the labeled target samples. Although achieving better performance in these applications, it still has some drawbacks in EEG analysis across subjects. First, DANN requires the samples between source and target domains to be balanced, while for cross-subject EEG analysis, the samples in source domain are usually much larger than those in target domain, which shows severe imbalance. Second, since significant difference of EEG signals exists across subjects, some source domain samples may be extremely inconsistent with the distribution of the target domain, which will cause “negative transfer” [19,20] and make the performance of DANN decrease in cross-subject EEG analysis.

In view of the shortcomings encountered by DANN in cross-subject EEG analysis, we propose an improved model, Generative-DANN (GDANN), by combining Generative Adversarial Networks (GAN) [21] to improve the ability in EEG analysis across subjects through the following three aspects: (a) the hidden layer, optimizer and loss function of DANN are improved to meet the requirement of cross-subject EEG analysis; (b) random noise is selected through GAN to generate sufficient fake data, which are close to the data distribution in the target domain, to balance the data set in the source and target domains to assist model training; and (c) when faced with multiple source domain data, GDANN can select the samples from the subjects with the closest data distribution to ensure cross-subject analysis performance more effectively and avoid negative transferring to a certain extent.

The rest of this paper is organized as follows. Section 2 introduces the process of the experiment. Section 3 elaborates the proposed model in detail. In Section 4 and Section 5, we present and discuss the experimental results. Finally, we conclude and give our prospects in the future in Section 6.

## 2. Experiments

### 2.1. Subjects

Informed consent was obtained from 13 healthy volunteers after the explanation of the study, which was approved by the local institutional ethical committee of University of Rome, La Sapienza (Rome, Italy). The group was selected with the aim to have a homogeneous sample in terms of age (26.8 ± 3.2 years old), and driving expertise (all the participants drove daily and regularly). All of them are asked to avoid alcohol the day prior to the measurements and caffeine 5 h before the experiment. The experiment was conducted following the principles outlined in the Declaration of Helsinki of 1975, as revised in 2008.

### 2.2. Experimental Protocol

**Figure 1:**
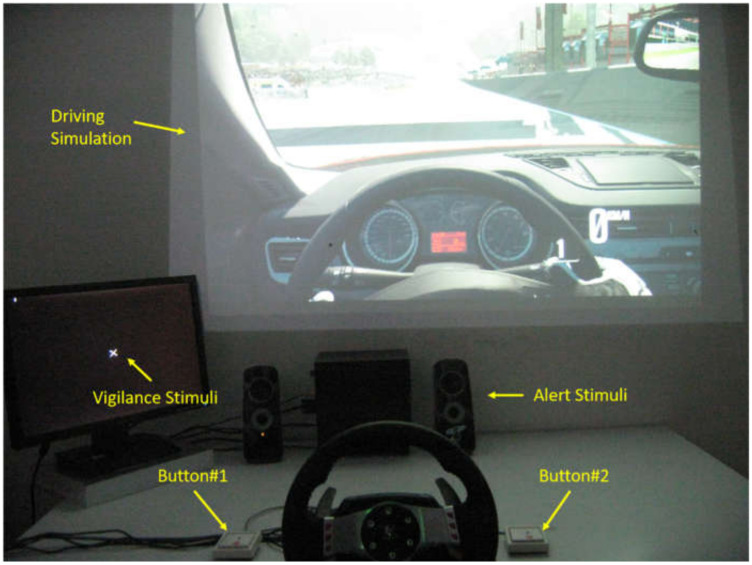
Experimental settings. (The experiment established five different Attentional and Vigilance tasks (TAV) to gradually increase the workload of the brain, allowing the subjects to drive a simulated car on the circuit under the same other conditions. When a white X appeared on the screen, subjects were asked to press Button#1, which represents “Vigilance Stimuli”. When the sound monitor emitted a stimulus, subjects were asked to press Button#2, which represents “Alert Stimuli”).

The experiments were performed within the same hours of the day to avoid bias in the results due to circadian rhythms or meals. In particular, the participants took part in the driving simulations between 2 PM and 5 PM. The simulation consisted in driving an Alfa Romeo—Giulietta QV (1750 TB*_i_*, 4 cylinders, 235 HP) on the Spa—Francorchamps (Belgium) track under different conditions. In order to modulate the difficulty of the driving simulation (i.e., primary task), the Attentional and Vigilance task (TAV) was employed as a secondary task [22,23]. When enabled, the TAV lasted for the entire corresponding experimental condition (i.e., 2 laps), and it worked as follows: the vigilance stimulus, a white X, was presented on the center of a monitor placed about 60 (cm) from the driver and below the main screen where the car cockpit was projected, as shown in Figure 1.

The drivers were asked to press the Button#1 as soon as the X appeared on the screen regardless of the ongoing driving situation. The vigilance stimuli simulates traffic-related events like red-turning traffic lights, road crossing pedestrians, other cars or other uncontrollable traffic agents. The acoustic alert stimulus was presented by two speakers placed on the left and right sides of the driver. A sequence of frequent (with a 95% probability rate) and target (5% probability) tones at different acoustic frequencies were continuously delivered to the drivers. They were asked to press the Button#2 as soon as the target stimulus occurred. The frequent acoustic tones simulated the car’s radio or engine noise, while the target ones reproduced unexpected events like a phone call. Five levels of the TAV were designed by setting different stimulation rates in order to induce different levels of workload demands and an overall mental fatigue status in the driver [3,24,25]. Before starting the experiment, the participants took part in a training session of half an hour to get familiar with the simulator commands and interface. Afterward, they initially drove the car through the selected track without any requests in terms of speed but always maintaining the car within the path. Such a condition was named “warm up” (WUP) and was aimed at defining the driver’s baseline. Then, the drivers are asked to repeat the 2 laps by driving as fast as they could but always by ensuring high safety, that, is avoiding driving off the path; this was the “performance” condition (PERFO). After the PERFO condition, the five TAVs (TAV1 to TAV5) were presented in a random order across the participants, where TAV1 was the easiest condition (i.e., very slow stimulation), whilst TAV5 was the most difficult one (i.e., very fast stimulation). Finally, the last experimental condition consisted of monotonous driving, in which the participants had to drive without exceeding the speed of 70 km/h. The purpose of the consecutive TAVs was to induce mental fatigue, while the last one aimed to make the drivers hypo-vigilant or drowsy (DROW). Except for the last stage of the DROW, which took 10 min, the remaining 7 stages in the experiment only took 2.5 to 4 min, so the total time required for the experiment was approximately 32–45 min. At the end of each experiment, the participants were asked to fill in the NASA-TLX questionnaire to provide the subjective workload perception [26]. In addition, errors in terms of keeping the car within the path and missed or wrong TAV answers were gathered.

### 2.3. EEG Recording and Preprocessing

EEG was recorded by a digital monitoring system (Brain Products GmbH, Munich, Germany) with a sampling frequency of 200 Hz. All 61 EEG channels were referred to both earlobes, grounded to the $FC_{z}$ channel, and their impedances were kept below 10 KΩ. After that, a band-pass filter was used for keeping EEG data between 1 Hz and 30 Hz, and Independent Component Analysis (ICA) [27] was adopted to remove the artifacts caused by eye blinking. After removing artifacts, we applied a 1 s sliding window with 10% overlap to segment EEG signals into 61 channels, and 400 segments of each condition were acquired for each subject.

**Figure 2:**
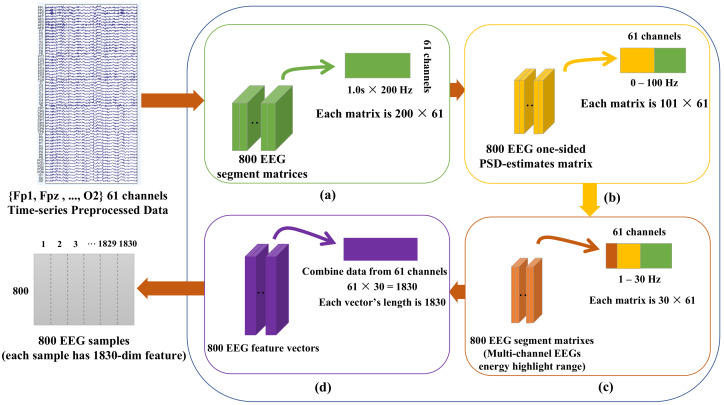
Schematic Diagram of electroencephalogram (EEG) power spectral density (PSD) extraction. ((**a**) shows that, we obtained 400 segment matrices for each condition regarding 61 EEG channels, and due to 200 Hz of sampling frequency and 1 s sliding window, the dimension of each matrix was 200 × 61; (**b**) reveals the one-side PSD estimate of the 100 Hz EEG signals, which means the logarithm of the signal power at an integer frequency between 0 and 100 Hz [29]. Regarding PSD-related frequency band, we chose the range from 0.1 to 30 Hz of EEG as the input signals (**c**). Each segment of the integer frequency between 0 and 30 Hz contained 30 EEG powers and 1830 powers for 61 channels. Correspondingly, the 400 × 1830 of dimensional feature vectors in each segment were obtained at the end, as shown in (**d**)).

Then, power spectral density (PSD), which is more sensitive in the range of frequency from 0.1 to 30 Hz [28,29], was chosen to transform three-dimensional time-series segments into two-dimensional sample data. The detailed schematic diagram of PSD is shown in Figure 2.

Therefore, for 13 subjects, 400 samples could be obtained for each condition (TAV1-TAV5, PERFO and DROW), and each sample had 1830 dimensional features. Herein, the two conditions of TAV3 and DROW were selected as positive and negative examples for subsequent experiments, which is explained in Section 4.1.

### 2.4. Domain Adaptation Learning

Domain Adaptation is a representative method in transfer learning, which uses source domain samples that contain rich information to improve the performance for classifying other target domain samples. In domain adaptation, the samples in target domain for testing usually have no or only a few labels, while those in the source domain for training are rich in supervised information. The source domain and the target domain often belong to the same type of task, but the sample distribution between them is different.

For the domain adaptation task, if a common feature representation space can be extracted between the source domain and the target domain, then, in this feature space, the classifier model learned from the source domain features can also be used on the features of the target domain. In particular, the domain adaptation task is often converted into the task of finding the common feature representation space, which is the domain-invariant feature [30]. If the domain invariant features can be learned by the model, the classifier can be trained by the obtained invariant features to make it effective for both the source domain and the target domain.

DANN and GDANN work as described above, which is described in detail in Section 3 later. The data used for training and testing the model comes from the feature samples extracted in Section 2.3.

### 2.5. Cross-Subject Cross-Validation and Evaluation Index

For cross-subject cross-validation, unlike the previous intra-subject experiments, we retained the data of one subject from the data set, used them as test data (i.e., target domain data), and summarized the data of the remaining 12 subjects together as training data (i.e., source domain data). Obviously, there is no intersection of data between the two domains. In addition, it should be noted that in this experiment, our model required some unlabeled data from the target domain to assist training. After the model was trained, we evaluated the recognition performance on the retained subject’s data and recorded the results. This process was repeated until each subject had been used at least once as a testing subject.

A confusion matrix was used to analyze obtained results, which used count values to summarize the number of correct and incorrect predictions and to subdivide them by category. The confusion matrix shows which part of the classification model will be confused when making predictions. It is this decomposition of the results that overcomes the limitations of using only classification accuracy. The binary classification analysis used in this experiment is shown in Table 1.

Herein, recall means, in all the samples that are actually 1 (positive samples), the probability of correct prediction. Precision means the probability of correct prediction in all samples with a prediction of 1 (positive sample). Accuracy is the proportion of the samples that are predicted correctly in all samples. Furthermore, F1Score [31,32] is the harmonic mean of Precision and Recall.

## 3. Method

### 3.1. The Existing DANN

**Figure 3:**
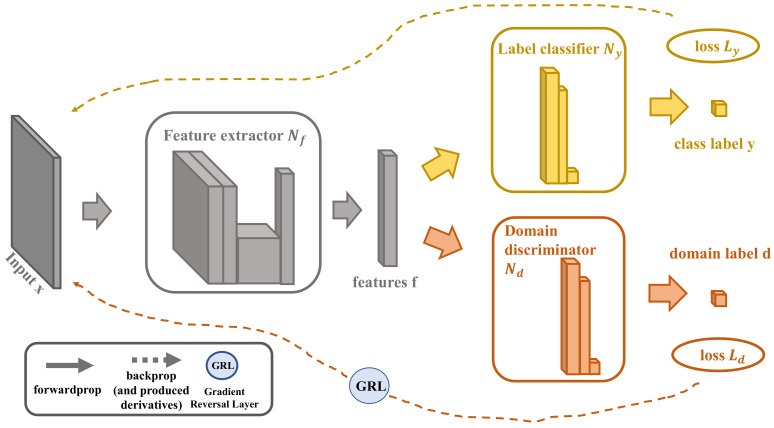
The Architecture of Domain-Adversarial Neural Network (DANN). (DANN combines three networks ($N_{f}$, $N_{y}$, and $N_{d}$) to align data from different distributions of source and target domains).

The architecture of DANN consists of three kinds of networks, as shown in Figure 3: (a) Feature Extractor Network $N_{f}\left(\cdot ;\mu_{f}\right)$, which adopts a shallow network model to extract the potential features with the parameter $\mu_{f}$ from both the source and the target domain; (b) Label Predictor Network $N_{y}\left(\cdot ;\mu_{y}\right)$, which is essentially a binary classifier and in charge of training the predictor with the labeled source domain data with the parameter $\mu_{y}$ and loss value $L_{y}$; and (c) Domain Classifier Network $N_{d}\left(\cdot ;\mu_{d}\right)$, which extracts the invariant features through the parameter $\mu_{d}$ and loss value $L_{d}$ and then judges whether the features come from the source domain or the target domain. During the training period of back-propagation, DANN implements unsupervised domain adaptation by adding a Gradient Reversal Layer (GRL) between $N_{f}$ and $N_{d}$ [18].

### 3.2. The Architecture of GDANN

**Figure 4:**
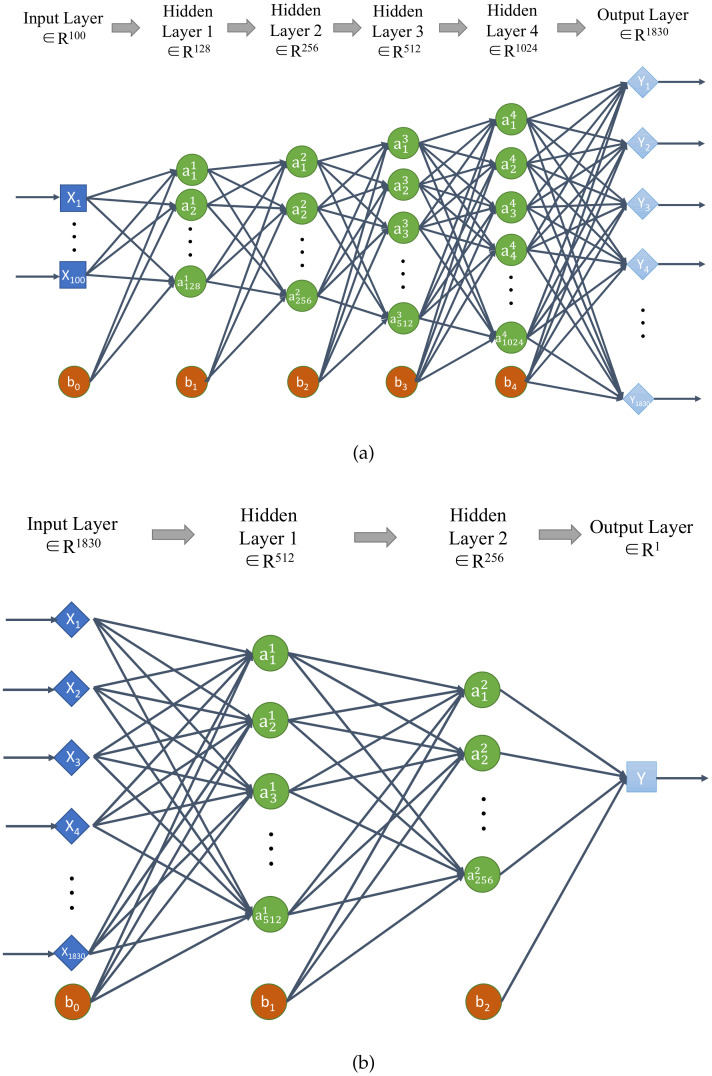
The network structure of Generative Adversarial Networks (GAN) ((**a**) $N_{tg}$ and (**b**) $N_{td}$).

On the basis of DANN, we added GAN networks, which consists of (a) the generator network $N_{tg}\left(\cdot ;\mu_{tg}\right)$, which is used to generate the fake target domain data with its parameter of $\mu_{tg}$ and loss value $L_{tg}$, and (b) the classifier network $N_{td}\left(\cdot ;\mu_{td}\right)$, which is used to identify the real target domain data and the fake target domain data with its parameter of $\mu_{td}$ and loss value $L_{td}$. The parameter μ should contain the weight *W* and the bias *b* of each layer network. Their network structure is shown in Figure 4, which includes the input layer, output layer, several hidden layers and the bias node.

For the improvement of all layers of GDANN, the LeakyRelu function was employed as the activation function, as shown in Equation (1), where leak∈(0,1) is a constant. In this way, some negatives are retained, and the correspondingly effective information will not be completely lost.

(1) LeakyRelu(α)=max(0,α)+leak×min(0,α)

The final output layer of the $N_{tg}$ and $N_{f}$ uses the Tanh function to output the result, which could effectively reduce the probability of saturation compared to the traditional Sigmoid function used in DANN [33]. In the newly added $N_{tg}$ and $N_{td}$ networks, the adaptive time estimation method (Adam function [34]) is used to perform the gradient descent, to make GDANN have better convergence. Meanwhile, $N_{f}$, $N_{y}$ and $N_{d}$ networks adopt SGD function [18] for parameter update optimization which is used by DANN.

As for the loss function of $L_{tg}$, $L_{td}$, $L_{d}$, and $L_{y}$, the CrossEntropy is used to predict the difference between the prediction data and true data. As shown in Equation (2), where *x* represents a sample, p(x) and q(x) are the true sample distribution and the predicted sample distribution of *x*, respectively.

(2) $$CrossEntropy\left(p,q\right)=-\sum_{x}p\left(x\right)\log q\left(x\right).$$

Thus, the network architecture of GDANN is combined with the three networks of DANN ($N_{f}$, $N_{y}$, and $N_{d}$), and GAN ($N_{td}$ and $N_{tg}$).

### 3.3. The Training Process of GDANN

We define *X* as the input space, Y=0,1…,L−1 as a collection of *L* labels. Therefore, data *D* can be expressed as $D={\left\{\left(x_{i},y_{i}\right)\right\}}_{i=1}^{n}\in R^{x*y}$, where x∈X,y∈Y. In this case, we take binary classifications as an example (0 for wakefulness and 1 for fatigue), i.e., L=2. Suppose that we have two different distributions on X∗Y, which are called the source domain $D_{s}$ (consisting of data from 12 subjects) and the target domain $D_{t}$ (consisting of data from the remaining subjects). The data distribution of the source domain and target domain are $P_{s}$ and $P_{t}$, respectively.

The goal is to train GDANN by using (1) the data on suitable $N_{s}$$\left(1\leq N_{s}<13\right)$ subjects in $D_{s}$, which is close to the distribution of $P_{s}$; (2) a certain proportion (denote λ as the scaling factor) of randomly selected unlabeled data $X_{t1}$ in $D_{t}$, which is line with the distribution $P_{t}$; and (3) the parameter μ, wherein we define $n_{s}$ as the number of samples in source domain data set, $n_{t}$ as the number of $X_{t}$ (i.e., 800), $n_{t1}$ as the number of the randomly selected unlabeled $X_{t1}$. Then we can get: $n_{t1}=\lambda n_{t}$ and $n_{s}=N_{s}\times 800$. In addition, random noise *Z* that conforms to Gaussian distribution $P_{z}$ is denoted as Equation (3) and is used to generate equivalent number of fake data compared with those of source data, where *r* is a random number.

(3) $$Z=\frac{1}{\sqrt{2\pi }}e^{\left(-\frac{r^{2}}{2}\right)}\in P_{z}$$

**Figure 5:**
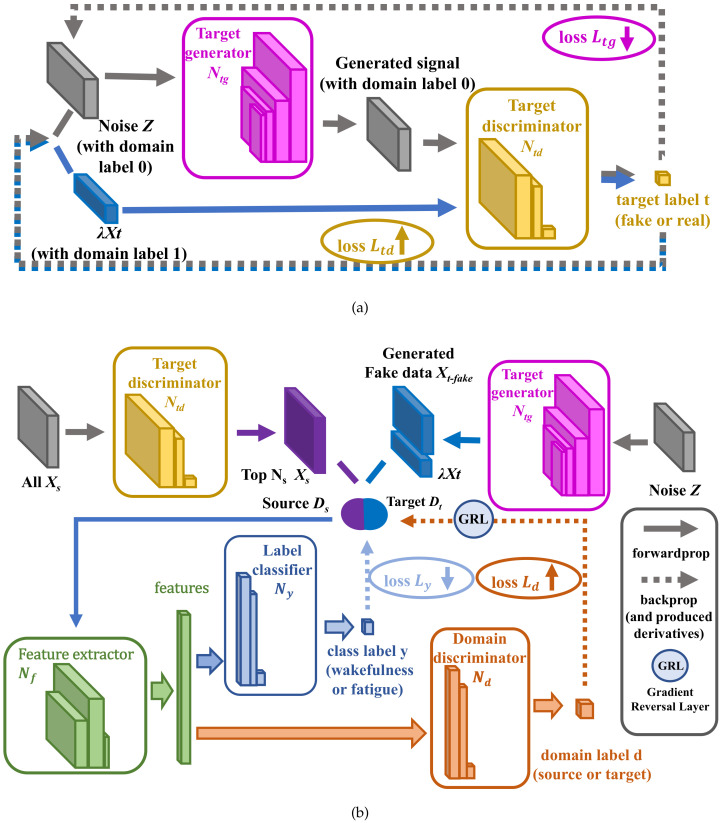
The Training Process of Generative-DANN (GDANN). ((**a**) is the process of training GAN with minimizing $N_{tg}$ and maximizing $N_{td}$, and (**b**) is the process of trainging DANN with minimizing $N_{f}$, $N_{y}$ and maximizing $N_{d}$, which contains the process of screening the top $N_{s}$ source domain subjects’ data that are most similar to the target domain and use random noise to train the domain alignment model together).

As shown in Figure 5, the training process of GDANN includes two steps: training GAN and training DANN. The design of this process embodies the idea of a two–two game and optimization, including $N_{td}$ vs. $N_{tg}$, and $N_{d}$ vs. $N_{f}$ and $N_{y}$. First, $N_{td}$ hopes to distinguish between real samples and fake samples as much as possible, while $N_{tg}$ tries its best to generate fake data conforming to the $P_{t}$ distribution to deceive $N_{td}$—this is a zero-sum game. Second, $N_{d}$ wants to distinguish the samples from the source domain and the target domain to the greatest extent, while $N_{f}$ extracts the domain features to fool $N_{d}$ for not discerning which domain those features come from—this is another zero-sum game. The optimization and competition of GDANN interplay with each other and finally reach the performance of global optimum, which will be shown through $N_{y}$ label classifier network.

As shown in Figure 5a, the first step is the confrontation between $N_{tg}$ and $N_{td}$. Their optimization process is expressed in Equations (4) and (5), which are the evolution of Equation (2). Here, noise *z* and the target domain data $X_{t1}$ (which are randomly selected from $X_{t}$ and the number is $n_{t1}$) are used to assist training. *Z* is the random noise (please see Equation (3)). The E(·) function means the optimization process, here is the process of maximizing $L_{td}$ and minimizing $L_{tg}$.

(4) $$\begin{array}{c} E\left(L_{tg}\right)=\min_{N_{tg}}L_{tg}\leftarrow \sum_{i=1}^{n_{t1}}\log\frac{1}{\sqrt[{n_{t1}}]{N_{tg}\left(Z_{i};\mu_{tg}\right)}} \end{array}$$

(5) $$\begin{array}{cc} E\left(L_{td}\right)=\max_{N_{td}}L_{td}\leftarrow \sum_{i=1}^{n_{t1}}\log & \left[\frac{1}{\sqrt[{2n_{t1}}]{\left(1-N_{td}\left(N_{tg}\left(Z_{i};\mu_{tg}\right);\mu_{td}\right)\right)}}\times \frac{1}{\sqrt[{2n_{t1}}]{N_{td}\left(X_{t1}^{i};\mu_{td}\right)}}\right] \end{array}$$

In an ideal state, the confrontation training between $N_{td}$ and $N_{tg}$ will reach the Nash equilibrium (i.e., $L_{td}=0.5$). At this time, $N_{tg}$ can generate fake data that conform to the $P_{t}$ distribution, and $N_{td}$ also has a certain ability to discriminate the true and fake data; that is, $N_{td}$ can roughly judge the similarity between the data and the $P_{t}$ distribution.

Then, the training process enters the second stage which is shown in Figure 5b. After the training of $N_{tg}$ and $N_{td}$, each subject’s data are passed through the $N_{td}$ network to obtain their distribution probability. Thus, $N_{s}$ subjects with the highest probability ranking are selected by the model from the $X_{s}$ data, which are closest to the $P_{t}$ distribution and good for transfer learning.

The training data on $N_{y}$ are derived from the domain features, which come from the top $N_{s}$ subjects’ data, and are generated through $N_{f}$. It is just a standard classifier based on neural network technology. In the process of the global optimization, the loss should be minimized, as shown in Equation (6) (the evolution of Equation (2)), where $n_{s}$ is the total number of $N_{s}$ subjects’ data, and $Y_{s}$ is the label of target domain’s feature matrix, i.e., $Y_{s}\in \left\{0,1\right\}$.

(6) $$E\left(L_{y}\right)=\min_{N_{f},N_{y}}L_{y}\leftarrow \sum_{i=1}^{n_{s}}\log[{\left(1-N_{y}(N_{f}(X_{s}^{i};\mu_{f});\mu_{y})\right)}^{\frac{Y_{s}^{i}-1}{n_{s}}}\times {\left(N_{y}(N_{f}(X_{s}^{i};\mu_{f});\mu_{y})\right)}^{\frac{-Y_{s}^{i}}{n_{s}}}]$$

The auxiliary fake data, $X_{t-fake}$, which also obey $P_{t}$ distribution in the target domain $X_{t}$, is generated by a large number of noise *Z* through $N_{tg}$, whose number is $\left(n_{s}-n_{t}\right)$; thus, it makes the number of sample data in $X_{t}$ ($n_{t}\ll n_{s}$, where $n_{s}$ is the number of data in $X_{s}$) be increased, so that the number of data in both domains is balanced. Then, a total of approximate $2n_{s}$ in both domains can be used for training, as shown in Equation (7) (the evolution of Equation (2)).

(7) $$E\left(L_{d}\right)=\max_{N_{d}}L_{d}\leftarrow \sum_{i=1}^{n_{s}}\log{\left(1-N_{d}(N_{f}(X_{s}^{i};\mu_{f});\mu_{d})\right)}^{\frac{-1}{n_{s}}}+\sum_{i=1}^{n_{t}}\log{\left(N_{d}(N_{f}(X_{t}^{i};\mu_{f});\mu_{d})\right)}^{\frac{-1}{n_{t}}}\;+\sum_{i=1}^{n_{s}-n_{t}}\log{\left(N_{d}(N_{f}(N_{tg}(Z_{i};\mu_{tg}^{*});\mu_{f});\mu_{d})\right)}^{\frac{1}{n_{t}-n_{s}}}$$

## 4. Results

This section presents the performance of the proposed approach on samples from the industry and neural science laboratory in University of Rome, La Sapienza’, which include the EEG recordings of 13 subjects. The work was executed on a computer with 16 GB RAM, NVIDIA GeForce GTX 1660 graphic memory with 6 GB, and Intel Core i5-9400F @ 2.9 GHz processor. Python 3.6.8 tools were adopted to verify GDANN algorithm under Linux environment with Ubuntu 5.4 operating system.

We usd four models, DANN, GDANN, Support Vector Machine (SVM) [35] and Easy Transfer Learning (EasyTL) [36], to perform the cross-subject cross-validation process, which was mentioned in Section 2.5. In addition, the obtained EEG data were pre-processed by the method of Section 2.3. The above process was repeated 5 times to get the average result and analyzed.

### 4.1. Selection of Regression Labels

**Figure 6:**
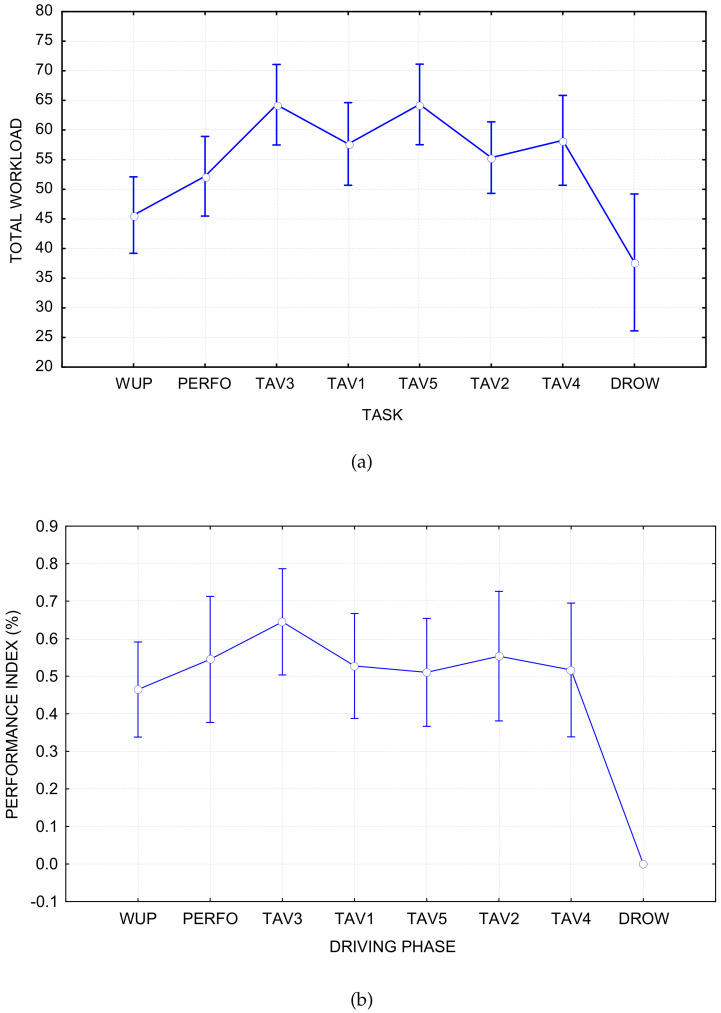
Reasons for the choice of TAV3 and DROW as the regressor labels. ((**a**) is the process of NASA-TLX (subjective) with $p<1\times 10^{-5}$, and (**b**) is the process of Behavioural (Performance) with $p<1\times 10^{-5}$).

The subjective (i.e., NASA-TLX) and performance (i.e., Driving Performance) data were used to identify the experimental conditions by which our deep-learning model was trained. In particular, the results reported in Figure 6 show that the TAV3 and DROW required, respectively, the lowest and highest cognitive demand; therefore, they were used as calibration data set. Our previous work [37,38] also proved that TAV3 and DROW are the two most different states, which are suitable for regression labels for fatigue state classification. This means that each subject (13 subjects in total) will get a total of 800 samples, of which 400 are from TAV3 with a label of 0, and the rest are from DROW with a label of 1.

### 4.2. Parameter Sensitivity

**Figure 7:**
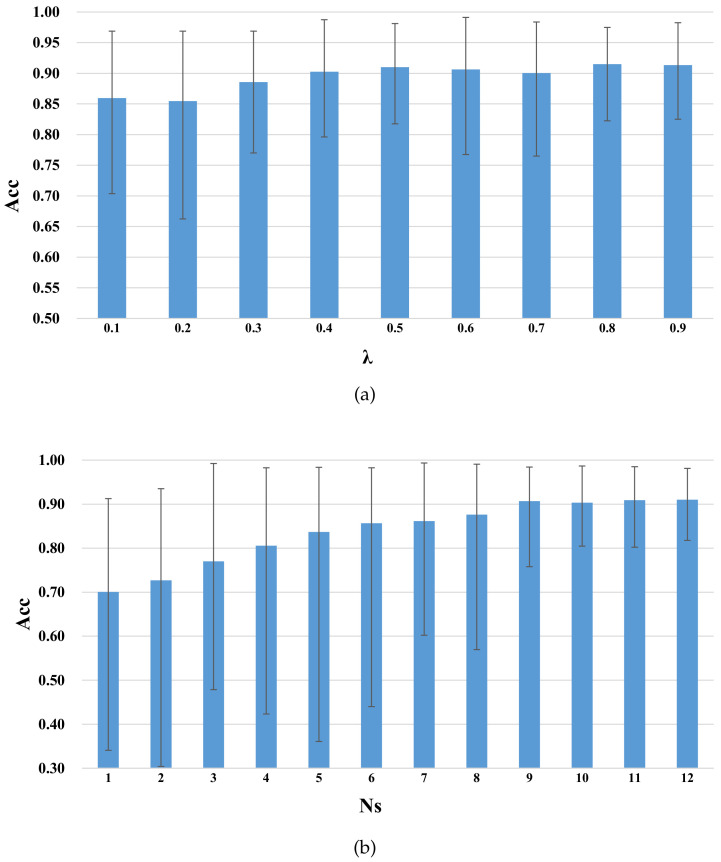
Parameter sensitivity of GDANN ((**a**) shows the parameter influence of λ with $N_{s}=12$, and (**b**) shows the parameter influence of $N_{s}$ with λ=0.5).

In order to obtain a more stable performance, we then studied the sensitivity of the parameters of λ (scaling factor, which was used to choose the samples of unlabeled $\lambda X_{t}$ from $X_{t}$ to assist training), $N_{s}$ (source subject number, which was used to select the best $N_{s}$ subjects from 13 to assist training) in GDANN. We then adopted a kind of grid search method to observe their sensitivity. That is, when optimizing one parameter, the other parameter remained unchanged, thus we could observe the optimal training performance. These two parameter values are determined from the following two ranges, which are: {0.1, 0.2, 0.3, 0.4, 0.5, 0.6, 0.7, 0.8, 0.9} for λ, and {1, 2, 3, 4, 5, 6, 7, 8, 9, 10, 11, 12} for $N_{s}$. The results are shown in Figure 7.

The “Acc” values in Figure 7 indicate that the average accuracy obtained by using the GDANN model for experiments under these parameter values (namely λ in Figure 7a, $N_{s}$ in Figure 7b), which can exclude the differences in model performance among different subjects. Choosing different values of λ, and $N_{s}$ has the following effects:

(a) In Figure 7a, as λ increases, the average accuracy of GDANN tends to increase slowly. To better reflect the robustness of the model, λ=0.5 was selected.
(b) In Figure 7b, as the number of source subjects increases from 1 to 9, the accuracy increases sharply. When the number reaches 9, the accuracy remains stable, and the accuracy curve may fluctuate slightly. Thus, we set $N_{s}=9$ in the following experiments.

In addition, the model also includes the following parameter settings: Epoch=50, Batch_Size=64, Adam_Learning_Rate=0.00001, Adam_b1=0.5, Adam_b2=0.999, SGD_Learning_Rate=0.005 and SGD_Momentum=0.9.

### 4.3. High-Dimensional Feature Visualization

**Figure 8:**
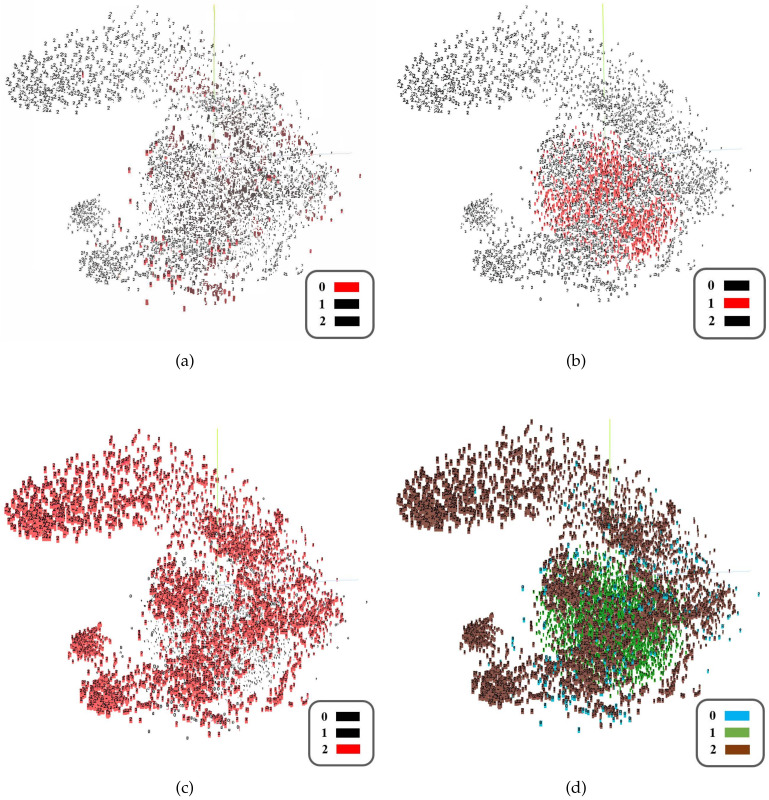
High-dimensional feature visualization results (t-SNE) on EEG DataSet Others_Subject#1. ((**a**) shows unlabeled target domain data with tag 0, (**b**) shows generated target domain data with tag 1, (**c**) shows source domain data with tag 2, and (**d**) is the collective display of data).

Since the training of the model relies on the auxiliary fake data from $N_{tg}$, we used the t-SNE method [39] to measure the quality of the data generated by GAN. For example, we took Subject#1 as the target domain data and used the default parameters of t-SNE; after 1000 iterations, the high-dimensional feature visualization results shown in Figure 8 were obtained. Here, tag 0 represents the unlabeled target domain data (Figure 8a), tag 1 represents the target domain data generated in GAN (Figure 8b), and tag 2 represents the source domain data (Figure 8c). It can be seen that the compact and dense generated fake data are roughly centered on the center of the unlabeled target domain data, while the distribution of the source domain data is relatively scattered and extensive.

### 4.4. Performance Comparison between GDANN and DANN

In order to reflect the improvement of the proposed model, we first compared the performance between GDANN and DANN, then using the optimal parameters obtained above, we analyzed the performance of the related indexes of Accuracy, Precision, F1Score and Recall for it. In our experiment, PSD (Power Spectral Density) was extracted as the input to train GDANN and DANN. The same Training Set and Testing Set were used for the purposes of training and testing, respectively. Table 2 shows the average results obtained after five times of repeated experiments.

#### 4.4.1. Statistical Analysis

**Figure 9:**
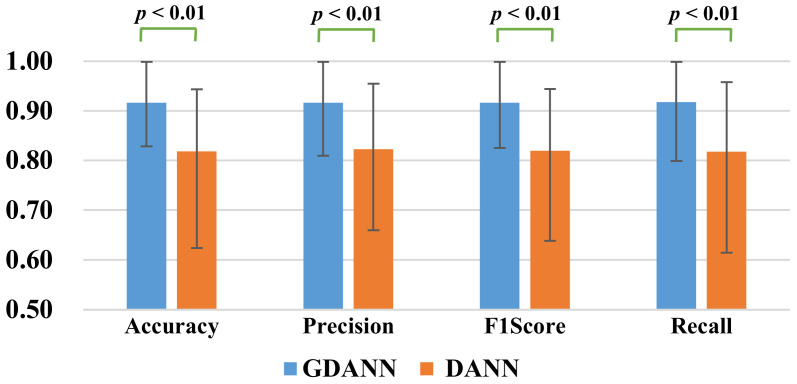
Paired Difference Analysis between four indicators of DANN and GDANN by Wilcoxon Signed Ranks.

For statistical validation of the results, a two-tailed Wilcoxon Signed Ranks Test [40] analysis was adopted to compare the performance of significant difference of Recall, Precision, Accuracy, and F1Score between DANN and GDANN using the data displayed in Table 2. The results of the two-tailed Wilcoxon Signed-Ranks Test are shown in Figure 9. The *p*-values of the four indicators are all less than 0.01, which means significant difference, validating the claim of better performance of GDANN.

#### 4.4.2. Convergency Analysis

**Figure 10:**
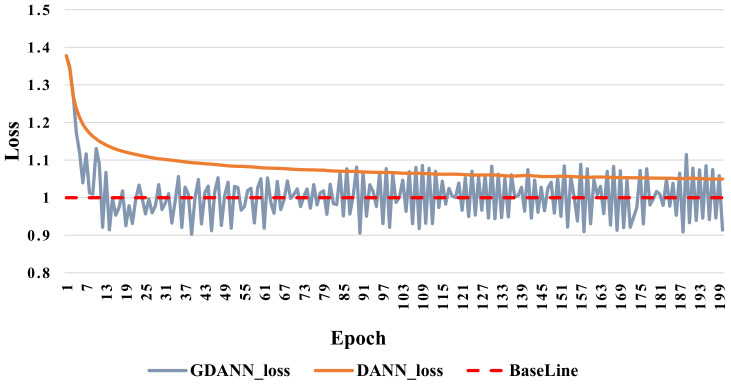
Loss of DANN and GDANN on EEG DataSet Others_Subject#1. (The Baseline refers to the saddle point where the training converged and reached the global optimal value, i.e., 1).

Additionally, the change in loss rate and convergence were analyzed between DANN and GDANN, as shown in Figure 10. When regarding the loss rate, we also take the Subject#1 as the target domain data and then observed the loss rate with the increase of the training epoch of the DANN and GDANN. Here, the X-axis represents the training epoch, and the Y-axis represents the total loss, which is the sum of the loss $L_{d}$ of the domain discriminator $N_{d}$ and the loss $L_{y}$ of the label classifier $N_{y}$. The Baseline here is 1, because this is the process of maximizing $L_{d}$ and minimizing $L_{y}$, and the ideal state of optimization is close to 1. Figure 10 shows that the total loss of GDANN and DANN changed significantly at the beginning, and then GDANN started to reach the baseline before DANN in the 15th epoch and fluctuated around the baseline; DANN reached balance in the 50th epoch, and the total loss stayed near 1.05; it was hard to continue training downwards.

### 4.5. Performance Comparison between GDANN and Other Existing Models

In order to verify the efficacy of the proposed method, we also compared its performance with SVM and EasyTL. These methods were used as recommended in the referenced papers, and these were chosen for comparison for the following reasons. (a) SVM is the most common and efficient supervised machine learning method, and it can be used to highlight the powerful performance of transfer learning. (b) EasyTL is another useful transfer learning method which can be used to compare performance with GDANN.

**Figure 11:**
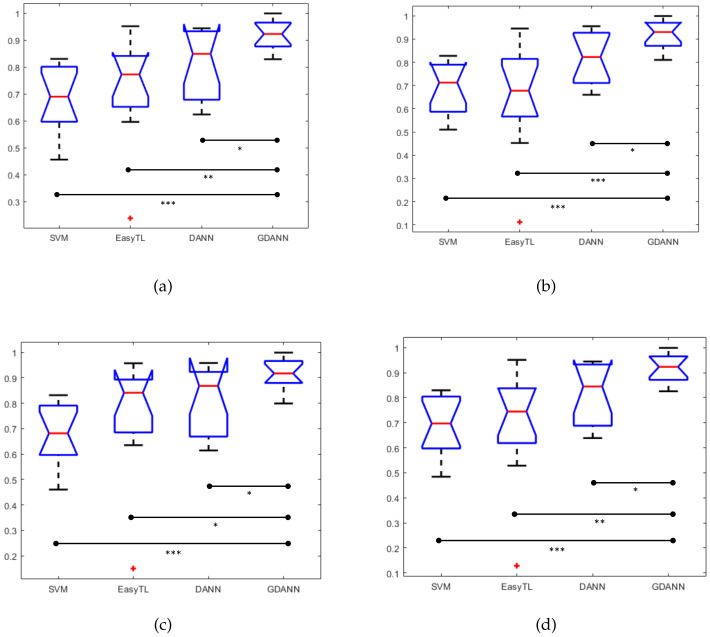
Box plots of confusion matrix ((**a**) Accuracy, (**b**) Precision, (**c**) Recall, and (**d**) F1Score) between SVM, EasyTL, DANN and GDANN. (* indicates *p* < 0.05; ** indicates *p* < 0.01; *** indicates *p* < 0.001).

Herein, it should be noted that in the process of comparison, EasyTL had the same training set and testing set as GDANN, while SVM did not need auxiliary training by unlabeled data but the training set from $X_{s}$ and testing set from $X_{t}$. We can get the corresponding box plots of the confusion matrix, as well as their paired t-test’s results, as shown in Figure 11. By comparing these four indicators, it can be seen that our model GDANN has a better performance than other three models. For example, in terms of accuracy, the improved model GDANN has an average accuracy of 91.63% and a performance improvement of 11.9% compared to the original model DANN, while EasyTL has an average accuracy of 72.95%, and SVM is 67.77%, reflecting the good performance of GDANN.

**Figure 12:**
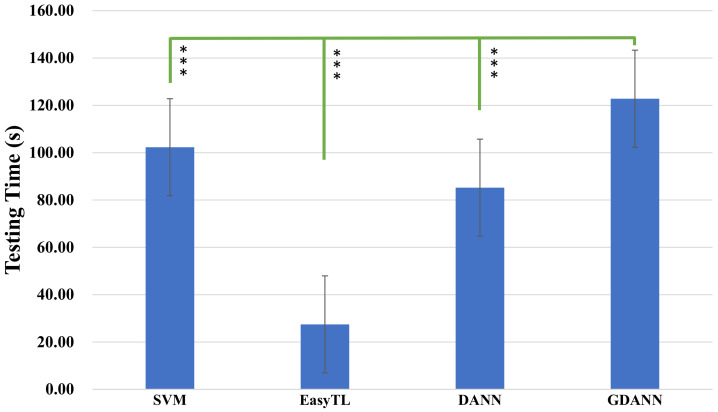
Testing time comparison between SVM, EasyTL, DANN and GDANN. (The testing time is the total time for one target subject to train the model and test results with the trained classifier, *** indicates *p* < 0.001).

Furthermore, we compared the testing time of the four models in Figure 12. The figure compares the average testing time required by the corresponding model, with positive and negative deviation error lines. It can be seen that GDANN (122.80 s) takes more time than SVM (102.3 s), EasyTL (27.44 s) and DANN (85.24 s).

## 5. Discussion

In the previous section, the proposed transfer learning model was been applied to data from the Industrial and NeuroScience Laboratory, University of Rome La Sapienza. Its performance was observed and compared with other standard classification techniques and transfer learning methods. The main observations of the results reported in Section 4 are as follows:

(a) Analysis of the number of subjects in the source domain: As analyzed in Section 4.2, in multi-source transfer learning, the source number $N_{s}$ is an important factor. More sources mean that we will integrate more data to predict fatigued driving. However, in view of the weak correlation between certain subjects, blindly increasing the number of sources may not improve accuracy and result in negative transfer and a calculation burden.
(b) Analysis of the generated target domain data: As analyzed in Section 4.3, the generated fake data roughly conform to the distribution of the target domain data, effectively making up for the shortcomings of insufficient training data.
(c) Comparison of the classification performance with the original model DANN: Due to the differences in the subjects, the classification performance of GDANN is also different. For those subjects who have good classification results in DANN, GDANN can give a slight improvement, while for those who do not perform well using the DANN method, GDANN will give a significant improvement. Since these accuracy values are not accidental (statistically verified), it can be said that a method for effectively performing EEG classification across subjects with multi-source training has been successfully proposed.
(d) Swiftly approaching convergence of baseline: In the convergence comparison, GDANN quickly reaches the baseline of loss training (i.e., 1) before DANN, and fluctuates on this line. Furthermore, DANN can only converge to 1.05 and cannot go down, which is also a manifestation of its insufficient performance.
(e) Comparison of classification accuracy with the state-of-the-art approaches: When comparing with some excellent related methods, such as supervised machine learning method SVM and transfer learning method EasyTL, as analyzed by Section 4.5, GDANN is seen to have better performance in terms of cross-subject EEG data prediction.
(f) Comparison of the testing time: In terms of testing time, the proposed work takes more time than other methods. It should be noted that compared to DANN, it has an additional process to adapt to generate auxiliary fake data. In practical use, the training epoch can be appropriately reduced to reduce the time. Most of the time is spent on training the model, and when the model training is completed, its high-efficiency performance can always be used.

## 6. Conclusions

In this paper, we propose an improved DANN-based transfer learning model, GDANN, and apply it for EEG-based cross-subject fatigue mental state prediction. Our GDANN model combines GAN with transfer model to make appropriate improvements and optimizations by balancing the difference in the number of samples between the source domain and the target domain, selecting the best Top N source domain subjects to experiment and extracting the invariant features of the target domain as much as possible. The transfer learning model can be conducted across different domain and data tasks. The experimental results show that the performance of GDANN is better than that of DANN, SVM and EasyTL. GDANN improves the EEG classification accuracy by about 11.9% with the original model DANN, which proposes a potentially powerful solution for fatigue state detection during driving.

In the future, we intend to enhance the simulation capabilities (by trying to cover all the edge data of the target domain) of GAN ($N_{tg}$ and $N_{td}$) to improve the performance of our model. Moreover, the size of the experimental group was not very big; therefore, one of the next steps could be to enlarge it to further validate the proposed method.

## Figures and Tables

**Table 1:** Binary confusion matrix indicator.

	Predicted = 1	Predicted = 0
Label = 1	TP (True Positive)	FP (False Positive)
Label = 0	FN (False Negative)	TN (True Negative)
1. Recall = TP/(TP + FN) 2. Precision = TP/(TP + FP) 3. Accuracy = (TP + TN)/(TP + TN + FN + TN) 4. F1Score = (2 × Precision × Recall)/(Precisioon + Recall)		

**Table 2:** Comparison results of the confusion matrix indicators between DANN and GDANN.

Others_Target Subject ID	Accuracy		Precision		F1Score		Recall	
	DANN	GDANN	DANN	GDANN	DANN	GDANN	DANN	GDANN
Others_Subject #1	0.6238	0.8294	0.6650	0.8800	0.6387	0.8376	0.6143	0.7991
Others_Subject #2	0.8838	0.9531	0.8900	0.9413	0.8845	0.9525	0.8790	0.9643
Others_Subject #3	0.6638	0.8831	0.6750	0.8425	0.6675	0.8782	0.6601	0.9171
Others_Subject #4	0.9363	0.9988	0.9550	0.9988	0.9374	0.9988	0.9205	0.9988
Others_Subject #5	0.8113	0.9019	0.7875	0.9150	0.8067	0.9032	0.8268	0.8916
Others_Subject #6	0.9313	0.9625	0.9325	0.9688	0.9313	0.9627	0.9302	0.9568
Others_Subject #7	0.6825	0.8831	0.7225	0.9038	0.6947	0.8840	0.6690	0.8695
Others_Subject #8	0.6663	0.8288	0.6600	0.8100	0.6642	0.8255	0.6684	0.8416
Others_Subject #9	0.9438	0.9719	0.9550	0.9725	0.9444	0.9719	0.9340	0.9713
Others_Subject #10	0.9375	0.9863	0.9150	0.9763	0.9361	0.9861	0.9581	0.9962
Others_Subject #11	0.7963	0.8556	0.7900	0.8200	0.7950	0.8503	0.8000	0.8831
Others_Subject #12	0.8488	0.9225	0.8225	0.9300	0.8447	0.9231	0.8681	0.9163
Others_Subject #13	0.9113	0.9356	0.9250	0.9500	0.9125	0.9365	0.9002	0.9235
Average	0.8182	0.9163	0.8227	0.9161	0.8198	0.9162	0.8176	0.9176

## Data Availability

The EEG data used to support the findings of this study are from the Industrial and NeuroScience Laboratory, University of Rome La Sapienza, and are restricted by the Ethics Committee of University of Rome La Sapienza, in order to protect SUBJECT PRIVACY. Data are available from the co-author: Gianluca Borghini (gianluca.borghini@uniroma1.it) for researchers who meet the criteria for access to confidential data.

## Abbreviations

The following abbreviations are used in this manuscript:

EEG	Electroencephalogram
DANN	Domain-Adversarial Neural Network
GDANN	Generative-DANN
GAN	Generative Adversarial Networks
SVM	Support Vector Machine
EasyTL	Easy Transfer Learning
BCI	Brain-computer Interaction
PSD	Power Spectral Density
TAV	the Attentional and Vigilance task
WUP	warm up
DROW	drowsy
ICA	Independent Component Analysis
NASA-TLX	National Aeronautics and Space Administration–Task Load Index
t-SNE	t-distributed Stochastic Neighbor Embedding
